# Tropical diseases in the context of climate change and emerging European transmission

**DOI:** 10.1371/journal.pntd.0014038

**Published:** 2026-02-26

**Authors:** Maciej Grzybek

**Affiliations:** Department of Tropical Parasitology, Institute of Maritime and Tropical Medicine, Medical University of Gdańsk, Gdynia, Poland; George Washington University Medical Center, UNITED STATES OF AMERICA

The term “tropical disease” has long been used to describe a heterogeneous group of infectious diseases historically concentrated in regions between the Tropics of Cancer and Capricorn. This designation, rooted in geographic convention rather than biological necessity, has shaped global health priorities, institutional structures, and scientific discourse for more than a century. However, accelerating climate change, ecological transformation, and increased global connectivity are fundamentally altering the spatial distribution of infectious diseases. As a result, several pathogens traditionally labelled as tropical are now establishing sustained transmission cycles in temperate regions, including Europe [1]. This epidemiological transition calls into question the continued scientific validity and public health utility of the term “tropical disease”.

One of the most compelling examples of this shift is the West Nile virus (WNV). Initially regarded as a pathogen confined to tropical and subtropical regions of Africa, the Middle East, and parts of Asia, WNV has become firmly established across much of Europe over the past two decades. Since the early 2000s, Europe has experienced repeated autochthonous outbreaks, with marked intensification after 2010 [2]. Multiple countries now report annual seasonal transmission, severe neuroinvasive disease, and associated mortality. Importantly, recent attribution studies demonstrate that climate change, through rising temperatures, extended vector activity seasons, and enhanced viral replication within mosquito vectors, has been a key driver of the increasing frequency and intensity of WNV outbreaks in Europe [3]. In this context, WNV can no longer be considered imported or exotic infection, but rather a standard component of the European infectious disease landscape.

West Nile virus is emblematic of a broader pattern affecting multiple vector-borne pathogens. Dengue virus, Chikungunya virus, and Zika virus have all caused autochthonous transmission in Europe, facilitated by the expanding geographic range of competent Aedes mosquito vectors. *Aedes albopictus* is now established in large parts of southern and central Europe, while *Aedes aegypti* has re-emerged in parts of the Mediterranean basin [4]. Climate-driven increases in temperature and humidity have enhanced vector survival, shortened extrinsic incubation periods, and increased transmission suitability in regions previously considered climatically unsuitable. Predictive models consistently project further northward expansion of these vectors and increased population exposure under current climate trajectories.

The northward spread of *Hyalomma marginatum* ticks further illustrates the inadequacy of a latitude-based definition of tropical diseases. *H. marginatum*, the principal vector of Crimean–Congo haemorrhagic fever virus (CCHFV) in Europe, was historically associated with arid and semi-arid regions of Africa, the Middle East, and southern Eurasia. In recent years, entomological surveillance and ecological modelling have documented the establishment and expansion of *H. marginatum* populations across southern and south-eastern Europe, with repeated detections extending into central and western regions [5]. While long-distance dispersal via migratory birds has been recognised for decades, growing evidence indicates that climate-driven increases in temperature and reduced winter mortality now permit local survival and potential population establishment. Notably, the current distribution of *H. marginatum* in Europe exceeds the known endemic range of CCHFV, creating ecological conditions conducive to further emergence of severe viral haemorrhagic fever within temperate regions [6].

These developments highlight a fundamental limitation of the term “tropical disease”: it conflates historical geographic distribution with intrinsic biological characteristics. From a pathogen or vector perspective, there is nothing inherently tropical about WNV or the dengue virus. Their transmission dynamics are governed by ecological suitability, vector competence, host availability, and social determinants of exposure, factors that are increasingly present well beyond the tropics. Climate change is effectively redrawing the global map of infectious disease risk, rendering static, latitude-based classifications scientifically obsolete.

The continued use of the term “tropical disease” has tangible consequences. It risks underestimating the public health threat posed by these infections in temperate regions, reinforcing the perception that they remain external or imported problems. This framing may delay investment in surveillance systems, diagnostic capacity, clinical training, and vector control in regions newly affected by sustained transmission. Moreover, it perpetuates an artificial divide between “global health” and “domestic public health”, despite the growing convergence of infectious disease risks across income levels and geographic regions.

For neglected tropical diseases in particular, the issue is not merely geographic expansion but conceptual inertia. Neglect is driven by social vulnerability, political marginalisation, and underinvestment, rather than latitude. As climate change exposes new populations to traditionally neglected pathogens, there is a risk that emerging disease burdens in temperate, high-income regions will fall outside established research and funding frameworks, or conversely, that endemic populations in historically affected regions will be deprioritised once diseases are no longer perceived as geographically confined.

The term “tropical disease” should be critically re-evaluated and, where appropriate, replaced by classifications that better reflect contemporary epidemiology. Alternative frameworks emphasising transmission ecology, climate sensitivity, vector dependence, and social determinants of risk may provide more accurate and operationally useful categories. Terms such as “climate-sensitive infectious diseases” or “vector-borne neglected diseases” shift the focus from historical geography to the mechanisms that drive emergence and persistence, facilitating more flexible and forward-looking public health responses [7].

As climate change continues to alter the distribution of pathogens, vectors, and host populations, infectious disease frameworks rooted in historical geography require systematic re-evaluation [8]. The redistribution of diseases traditionally labelled as tropical into temperate regions underscores the need for terminology that reflects contemporary transmission ecology rather than legacy classifications. Redefining what is meant by a “tropical disease” is not a semantic adjustment, but a necessary response to measurable epidemiological change. Aligning scientific language with observed patterns of emergence and persistence is essential for accurate risk assessment, effective surveillance, and informed public health decision-making across regions with differing historical disease burdens.
